# Zea Maize Calmodulin (*ZmCaM2*) Regulates Drought Tolerance in Corn Plants Through an Abscisic Acid-Dependent Signaling Pathway

**DOI:** 10.3390/plants14233656

**Published:** 2025-11-30

**Authors:** Meiyi Liu, Pengxiang Bao, Hanqiao Wang, Zhiqiang Wu, Zhen Wang, Xiangyu Xing, Wei Yang, Xuejiao Ren, Jiabin Ci, Liangyu Jiang, Zhenyuan Zang

**Affiliations:** 1 College of Agriculture, Jilin Agricultural University, Changchun 130118, China; l2932144311@163.com (M.L.); 15193294617@163.com (P.B.); wanghanqiao02@163.com (H.W.); 17396774079@163.com (Z.W.); wangzhen021010@163.com (Z.W.); xxy17390949953@126.com (X.X.); davidyoung588@126.com (W.Y.); rxj0342@163.com (X.R.); cjb6666@163.com (J.C.); 2 Xiangzhou District Talent Development Center, Xiangyang 441000, China

**Keywords:** *ZmCaM2*, maize, drought, ABA, signaling pathway

## Abstract

Calmodulins (CaMs), which are important calcium-binding proteins, play critical roles in plant stress responses. However, limited information is available regarding the biological functions of CaMs under drought stress. In this study, we identified and isolated a CaM gene, *ZmCaM2*, from maize (*Zea mays* L.) in length and encodes a 184-amino acid protein containing four EF-hand domains capable of specifically binding calcium ions (Ca^2+^). Subcellular localization analysis revealed that ZmCaM2 is localized to the nucleus and membrane. Functional characterization indicated that *ZmCaM2* negatively regulates drought tolerance in maize by increasing malondialdehyde (MDA) and reactive oxygen species (ROS) content while decreasing antioxidant enzyme activity, proline (Pro) content, abscisic acid (ABA) content and relative water content (RWC). Moreover, *ZmCaM2* reduced maize sensitivity to ABA treatment, suggesting that *ZmCaM2* negatively regulates the drought tolerance of maize by relying on the ABA pathway. These findings provide new insights into the functional role of *ZmCaM2* and may facilitate the development of drought-resistant maize cultivars.

## 1. Introduction

With global warming and the increasing frequency of extreme weather events, drought stress has become a major factor limiting crop growth, development, and production [1,2]. For instance, severe drought from 1980 to 2015 reduced the yields of wheat and maize by 21% and 40%, respectively [3]. Drought stress caused approximately USD 30 billion in agricultural losses over the past two decades [4]. Thus, enhancing crop drought tolerance is a crucial goal for ensuring global food security. Zea maize (*Zea mays* L.), one of the world’s most important crops, serves as a key source of food, animal feed, and industrial raw materials. Its growth and development are severely affected by drought stress, resulting in yield losses of 30–90% [5]. Therefore, applying molecular breeding strategies to enhance drought tolerance is essential for sustainable maize production.

Calcium ions (Ca^2+^), as important second messengers, play vital roles in regulating various abiotic stress responses and in plant growth and development [6,7]. External stimuli can induce rapid changes in cytosolic Ca^2+^ concentration. Ca^2+^ binds to Ca^2+^-binding proteins (also known as Ca^2+^ sensors), thereby mediating signal transduction and triggering specific cellular responses [8]. Ca^2+^ sensors can be divided into several groups, such as calmodulin (CaM), calmodulin-like proteins (CMLs), calcium-dependent protein kinases (CDPKs), calcineurin B-like proteins (CBLs), and calcium and calmodulin-dependent protein kinases (CCaMKs) [9,10,11]. CDPKs and CCaMKs act as sensor–responder proteins, while CaMs, CMLs, and CBLs serve as sensor–relay proteins [12,13]. CaMs lack additional functional domains and inherent enzymatic activities [14]. CaMs have been extensively identified in multiple plant species. Specifically, 7 *CaMs* exist in *Arabidopsis* [15], 5 in *Oryza sativa* [16], 25 in *Brassica napus* [17], 4 in *Chrysanthemum seticuspe* [18], 5 in barley [19], 18 in wheat [20], and 3 in *Vitis vinifera* [21]. These proteins are highly conserved and represent some of the most ubiquitous Ca^2+^ sensors in eukaryotic organisms. Structurally, each CaM contains four canonical EF-hand motifs [22].

Growing evidence suggests that *CaMs* play essential roles in regulating plant growth and development, as well as in orchestrating responses to diverse abiotic and biotic stresses. The overexpression of *GmCaM4* enhances salt tolerance via increased DNA-binding activity of *MYB* [23]. Overexpression of *GmCaM4* improves soybean resistance to pathogens through activation of pathogenesis-related genes [24]. Similarly, overexpression of *OsCaM1-1* in rice enhances tolerance to salt and heat stress [25,26]. The *OsCaM1* mediated *CCaMK-MKK1/6* cascade plays a positive role in regulating lateral root growth in rice [27]. In *Arabidopsis*, *AtCaM1* and *AtCaM4* increase salt tolerance by promoting nitric oxide (NO) accumulation [28]. *AtCaM1*, as a positive regulator, participates in leaf senescence, abscisic acid (ABA) signaling, and reactive oxygen species (ROS) accumulation [29]. *AtCaM4* interacts with *PATL1* and reduces freezing tolerance in *Arabidopsis* by modulating the expression of *KIN1*, *COR15b*, and *COR8.6* [30,31]. Furthermore, *AtCaM4* negatively regulates ROS accumulation in the wound-signaling pathway through activation of *AtMPK8* [30]. *AtCaM3* regulates the heat shock signaling pathway, and its overexpression enhances thermotolerance in *Arabidopsis* [32]. The overexpression of *CaMs* enhanced the plant’s tolerance to low temperatures [33]. Additionally, Ca^2+^/CaM2 interacts with CYCLIC NUCLEOTIDE GATED CHANNEL 15 (*CNGC15s*) to maintain calcium oscillatory signaling and promote root nodule symbiosis in *Medicago truncatula* [34]. *CsCaM3* enhances cucumber tolerance to high-temperature stress by increasing the activity of antioxidant enzymes superoxide dismutase (SOD) and peroxidase (POD), and regulating the expression of genes involved in the chlorophyll biosynthesis and ABA signaling pathways [35]. Overexpression of *StCaM2* reduces the accumulation of ROS in tobacco, thereby enhancing its tolerance to drought and salt stress [36]. *HvCaM1* silencing lines exhibit increased tolerance to salt stress [37].

The plant hormone ABA plays a central role in regulating plant responses to abiotic and biotic stresses [38,39]. Accumulating evidence indicates that Ca^2+^ sensors are integral components of the ABA signaling pathway [40]. *CML37*, *CML38*, and *CML39* are transcriptionally induced by salt, drought, and ABA [41]. *AtCML9* negatively regulates salt tolerance through the ABA-mediated signaling pathway [42]. *AtCML20* functions as a negative regulator of drought tolerance through ABA-dependent pathways [43]. *EcCaM* overexpressing *Arabidopsis* exhibits hypersensitivity to ABA during seed germination and enhanced tolerance to drought and salinity stress [44]. Collectively, Ca^2+^ and ABA signaling constitute an intricate regulatory network controlling plant responses to abiotic stress.

Previously, we identified *CaM* genes in maize and observed that *ZmCaM2* can respond to drought stress based on transcriptome analysis [45]. However, the function of *ZmCaM2* in response to drought stress remains unclear. In this work, we conducted a detailed characterization of *ZmCaM2*, including subcellular localization and Ca^2+^-binding analyses, and elucidated its function in maize during drought stress. These findings lay the groundwork for the application of molecular breeding strategies to develop drought-tolerant maize cultivars.

## 2. Results

### 2.1. Cloning and Analysis of the ZmCaM2 Gene

**Figure 1:**
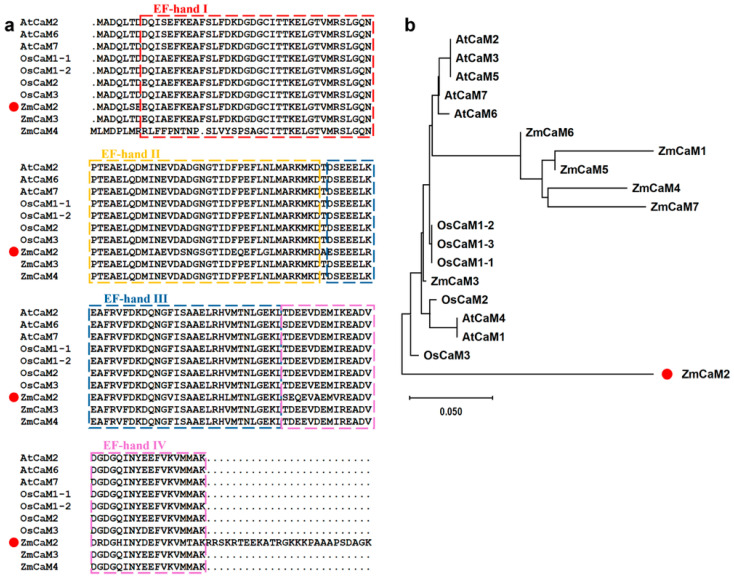
Multiple sequence alignment and phylogenetic analysis of ZmCaM2. (**a**) Alignment of amino acid sequences of the ZmCaM2 and other CaM proteins from *Arabidopsis* and rice, which the EF-hand motifs are underlined; (**b**) Phylogenetic tree analysis of ZmCaM2 and other orthologs in different species. The accession numbers are as follows: AtCaM1 (AT5G37780), AtCaM2 (At2g27030), AtCaM3 (At3g56800), AtCaM5 (At2g41110), AtCaM6 (At5g21274), AtCaM7 (At3g43810), OsCaM1-1 (LOC_Os03g20370), OsCaM1-2 (LOC_Os07g48780), OsCaM1-3(LOC_Os01g16240), OsCaM2 (LOC_Os05g41210), OsCaM3 (LOC-_Os01g17190), ZmCaM1 (Zm00001d028948), ZmCaM2 (Zm00001d043144), ZmCaM2-1 (Zm00001d040323), ZmCaM3 (Zm00001d038543), ZmCaM4 (Zm00001d038545), ZmCaM5 (Zm00001d022546), ZmCaM6(Zm00001d008278), ZmCaM7 (Zm00001d047597).

A 552 bp coding sequence (CDS) region was cloned from the maize inbred line B73 and designated as *ZmCaM2* (Zm00001d043144). ZmCaM2 encodes a protein of 184 amino acids, with a molecular weight of 20.47 kDa and a theoretical isoelectric point (pI) of 4.84. The gene is located on chromosome 3 and contains no predicted signal peptide. The negative grand average of hydropathicity (GRAVY) value (−0.743) suggests that ZmCaM2 is a hydrophilic protein. Multiple sequence alignment showed that ZmCaM2 contains four conserved EF-hand domains. Phylogenetic analysis indicated that ZmCaM2 is most closely related to OsCaM3 (Figure 1a,b).

### 2.2. ZmCaM2 Is Involved in the Response to PEG and ABA Induced Stress

**Figure 2:**
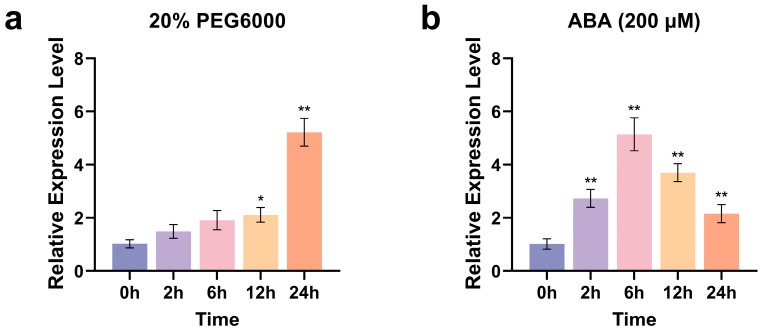
Expression patterns of *ZmCaM2* in response to 20% PEG6000 and 200 µM ABA treatments. (**a**,**b**) The relative expression levels were analyzed using the 2^−ΔΔCT^ method. The 0 h time point was set as the control. Statistical significance compared with 0 h was determined using one-way ANOVA (* *p* < 0.05, ** *p* < 0.01). The data are presented as means ± standard deviation (SD). Bars indicate the standard error of the mean. The experiment was performed using three biological replicates from three independent plants.

To verify the involvement of *ZmCaM2* in polyethylene glycol (PEG) and ABA-induced stress responses, uniformly grown maize seedlings at the third-leaf stage (V3 stage) were treated with 20% PEG 6000 (irrigation) or 200 µM ABA (foliar application). The transcriptional level of *ZmCaM2* in maize leaves was quantified by quantitative real-time polymerase chain reaction (qRT-PCR) under treatment with 20% PEG6000 and 200 µM ABA. Under 20% PEG6000-simulated drought stress, *ZmCaM2* expression remained slightly above the untreated control during the early stages, increased markedly after 12 h, and reached a maximum (5.2-fold) at 24 h (Figure 2a). Treatment with 200 µM ABA significantly induced *ZmCaM2* expression, which peaked at 6 h (5.1-fold) (Figure 2b). These findings suggest that *ZmCaM2* plays a critical role in regulating plant tolerance to drought stress and in the response to the ABA signaling pathway.

### 2.3. ZmCaM2 Is Localized to the Nucleus and Plasma Membrane and Can Bind Ca^2+^

**Figure 3:**
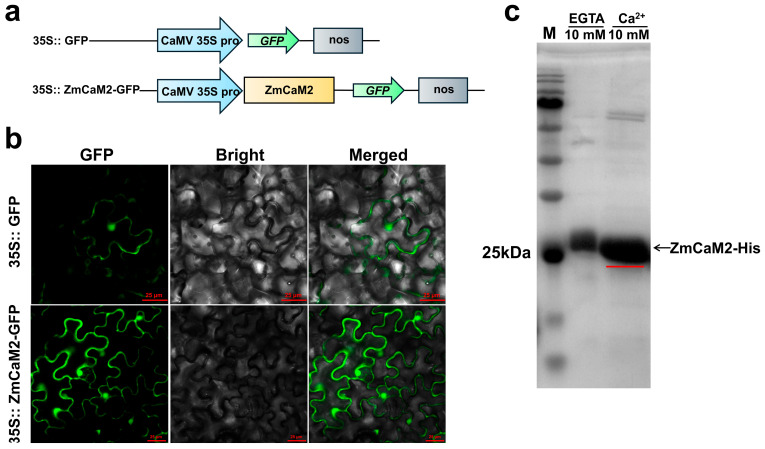
Subcellular localization of ZmCaM2 and Ca^2+^ binding properties. (**a**) Schematic diagram of pCAMBIA1302-GFP (35S::GFP) and pCAMBIA1302-ZmCaM2-GFP (35S:: ZmCaM2-GFP); (**b**) Subcellular Localization of the 35S:: ZmCaM2-GFP recombinant protein. The pCAMBIA1302-GFP vector was utilized as a control. Scale bars = 25 µm; (**c**) Ca^2+^ binding assay of ZmCaM2-His recombinant protein. The red line indicates the migration rate of the ZmCaM2-His recombinant protein.

For investigating the subcellular localization of the ZmCaM2, the pCAMBIA1302-ZmCaM2-green fluorescent protein (GFP) (35S::ZmCaM2-GFP) recombinant vector was introduced into *Nicotiana benthamiana* leaves. The pCAMBIA1302-GFP (35S::GFP) vector was used as a control. As depicted in Figure 3b, the 35S::GFP protein was expressed in both the nucleus and the cell membrane. Similarly, 35S::ZmCaM2-GFP was localized to both the nucleus and the membrane.

In order to verify whether ZmCaM2 has the ability to bind to Ca^2+^, the recombinant protein ZmCaM2-His was purified and then added to either 10 mM CaCl_2_ or 10 mM EGTA. As depicted in Figure 3c, the migration rate of the recombinant protein ZmCaM2-His was slower in the 10 mM EGTA solution compared to that in the Ca^2+^ solution. These results were consistent with the report by Garrigos, M. et al. (1991) [46], suggesting that ZmCaM2 is able to bind to Ca^2+^ in vitro.

### 2.4. ZmCaM2 Negatively Regulates Drought Tolerance in Maize

To validate the function of *ZmCaM2* in maize, we generated *ZmCaM2* overexpressing lines (OE1 and OE2) and CRISPR/Cas9-*ZmCaM2* mutant lines (C1 and C2). The T3 generation overexpressing lines were confirmed by bar strips and qRT-PCR (Figure S1), while the T3 generation CRISPR/Cas9-*ZmCaM2* mutants were verified by sequencing (Figure S2).

**Figure 4:**
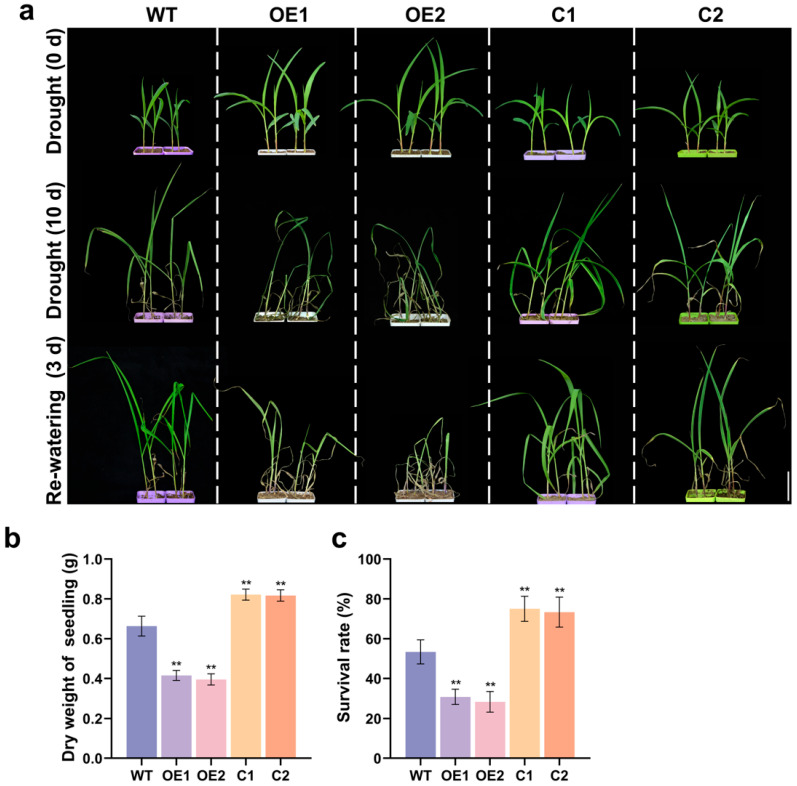
(**a**) Images of drought tolerance evaluation in WT, *ZmCaM2* overexpressing lines (OE1 and OE2), and CRISPR/Cas9-*ZmCaM2* mutant lines (C1 and C2) under drought treatment. Plants were photographed at 0 d, after 10 d of drought treatment (withholding water), and after 3 d of rewatering. Bar = 5 cm; (**b**) After 10 d of drought stress, whole-plant dry weight was measured in WT, *ZmCaM2* overexpressing, and CRISPR/Cas9-*ZmCaM2* mutant lines; (**c**) Survival rates of WT, overexpressing lines, and mutant lines after 3 d of rewatering. Each biological replicate contained 20 plants, and the survival rate of each replicate was calculated as the mean of 20 plants. WT was used as the control. The data are presented as means ± standard deviation (SD). Bars indicate the standard error of the mean. Statistical significance was determined using one-way ANOVA (** *p* < 0.01). The photographs show representative plants.

For drought tolerance evaluation, wild-type (WT) B104, overexpressing lines, and CRISPR/Cas9-*ZmCaM2* mutant lines were subjected to drought stress by withholding water for 10 days (d), followed by re-watering for 3 d. Following treatment, the overexpressing lines exhibited more severe wilting than WT, whereas the mutant lines showed less wilting (Figure 4a). The dry weight of the OE1 and OE2 lines was significantly lower than that of the WT, C1, and C2 strains, whereas the dry weight of the C1 and C2 lines was significantly higher than that of the WT (Figure 4b). After re-watering, the survival rates of OE1 and OE2 were 31% and 33%, respectively. WT, C1, and C2 showed survival rates of 52%, 75%, and 76%, respectively (Figure 4c). These results indicate that *ZmCaM2* negatively regulates drought tolerance in maize.

### 2.5. ZmCaM2 Decreases Maize Drought Tolerance by Affecting Antioxidant Enzyme Activity

**Figure 5:**
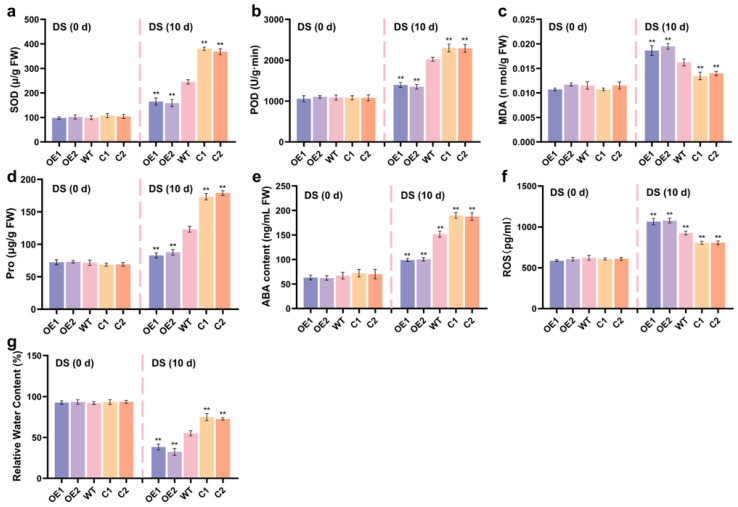
The physiological indices of the *ZmCaM2* overexpressing lines, CRISPR/Cas9-*ZmCaM2* mutant lines, and WT were investigated under drought stress conditions. (**a**) SOD activity; (**b**) POD activity; (**c**) MDA content; (**d**) Pro content; (**e**) ABA content; (**f**) ROS content; (**g**) RWC. Each experiment was performed with three biological replicates. The data are presented as means ± standard deviation (SD). The significance analysis compared with WT was conducted using one-way analysis of variance (ANOVA) (** *p* < 0.01). WT is set as the control. Bars indicate the standard error of the mean. The experiment was performed using three biological replicates from three independent plants. DS: drought stress.

To further investigate the physiological role of *ZmCaM2* under drought stress, the activities of SOD and POD, malondialdehyde (MDA) content, proline (Pro) content, ABA content, ROS content, and relative water content (RWC) were assessed in *ZmCaM2* overexpressing lines, CRISPR/Cas9-*ZmCaM2* mutant lines, and WT plants. Under normal conditions, no significant differences were observed in SOD and POD activity, MDA content, Pro content, ABA content, ROS content and RWC among the three genotypes (Figure 5a–g). After 10 d of drought treatment, SOD and POD activities in the OE1 and OE2 lines were lower than in the WT, whereas in the C1 and C2 lines, they were significantly higher than in the WT (Figure 5a,b). In the OE1 and OE2 lines, MDA and ROS content was significantly higher than in the WT lines. In contrast, MDA and ROS content in the C1 and C2 lines was significantly lower than in the WT lines (Figure 5c,f). As shown in Figure 5d,e, Pro and ABA content in the OE1 and OE2 lines was significantly lower than in the WT and C1 and C2 lines. Furthermore, Pro and ABA content in the C1 and C2 lines was significantly higher than in the WT lines. After drought treatment, RWC decreased across all maize lines. The OE1 and OE2 lines showed significantly lower RWC than the WT and C1 and C2 lines, whereas the C1 and C2 lines exhibited significantly higher RWC than the WT lines (Figure 5g). The level of relative water content serves as an indicator of a crop’s resistance to drought stress, with crops exhibiting higher relative water content demonstrating greater tolerance to such conditions. These findings suggest that *ZmCaM2* negatively regulates maize tolerance to drought stress by increasing MDA and ROS accumulation, reducing antioxidant enzyme activity, lowering ABA content, and decreasing RWC.

### 2.6. ZmCaM2 Negatively Regulates ABA Signal Transduction Pathway

**Figure 6:**
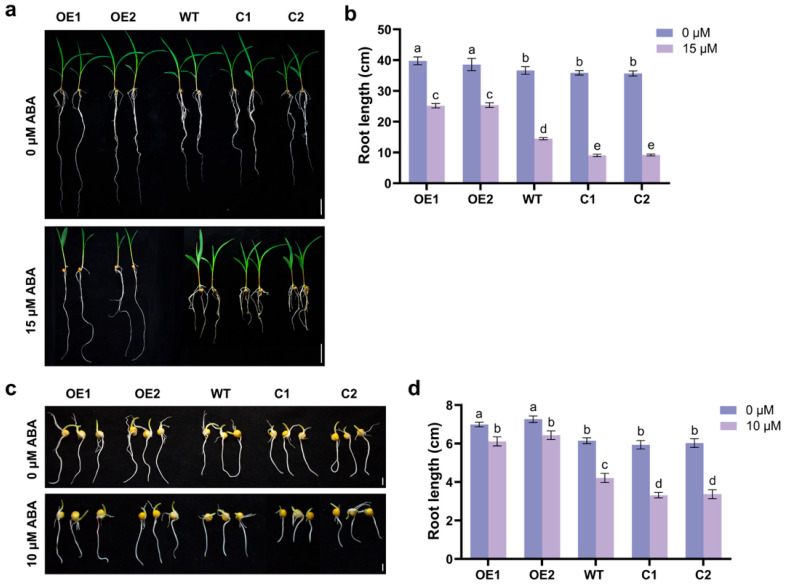
Physiological and molecular responses of maize to ABA in *ZmCaM2* lines (**a**) The phenotype of WT, *ZmCaM2* overexpressing lines (OE1 and OE2), and CRISPR/Cas9-*ZmCaM2* mutant lines (C1 and C2) grown in Hoagland solution with 0 µM ABA or 15 µM ABA. Scale bars = 5 cm. (**b**) The primary root length of WT, *ZmCaM2* overexpressing lines, and CRISPR/Cas9-*ZmCaM2* mutant lines were analyzed under 0 µM ABA or 15 µM ABA treatment. (**c**) Seed germination phenotypes of *ZmCaM2* overexpressing lines, CRISPR/Cas9-*ZmCaM2* mutant lines, and WT plants in 0 µM ABA or 10 µM ABA. Scale bar = 1 cm. (**d**) The primary root length of WT, *ZmCaM2* overexpressing lines, and CRISPR/Cas9-*ZmCaM2* mutant lines were analyzed under 0 µM ABA or 10 µM ABA treatment. Each experiment was performed with three biological replicates. The data are presented as means ± standard deviation (SD). The significance analysis was performed using two-way ANOVA (different lowercase letters indicate a difference at the 0.01 level *p* < 0.01). Bars indicate the standard error of the mean. The experiment was performed using three biological replicates from three independent plants.

To investigate the role of *ZmCaM2* in ABA signaling, WT, *ZmCaM2* overexpressing, and CRISPR/Cas9-*ZmCaM2* mutant seedlings were treated with 0 µM ABA or 15 µM ABA. The primary root length was used to assess the sensitivity of the three strains to ABA. The primary root length was measured on the tenth day. The results indicate that, compared with WT lines, C1 and C2 lines showed shorter primary root length under ABA treatment, whereas OE1 and OE2 lines had longer primary root length (Figure 6a,b). To further validate the *ZmCaM2* response to the ABA signaling pathway, we performed germination assays on WT seeds, *ZmCaM2* overexpressing seeds, and CRISPR/Cas9-*ZmCaM2* mutant seeds treated with 0 μM ABA or 10 μM ABA. The primary root length was measured on the fourth day. Results indicate that the C1 and C2 lines had significantly greater shorter primary root length than the WT lines under ABA treatment, while the OE1 and OE2 lines had significantly longer primary root length than the WT lines (Figure 6c,d). These results indicate that the overexpression of *ZmCaM2* reduces maize sensitivity to ABA stress.

**Figure 7:**
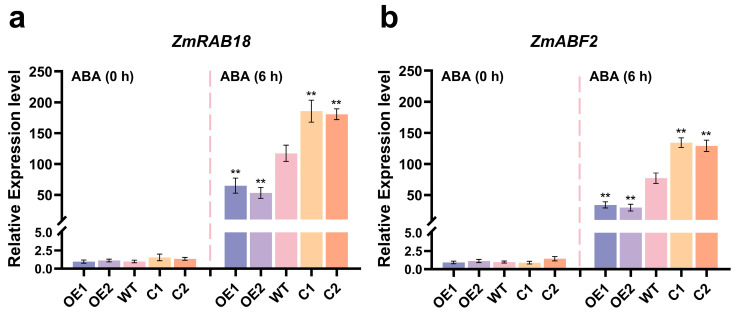
The relative expression levels of ABA-responsive genes, including (**a**) *ZmRAB18* (GRMZM2G098750) and (**b**) *ZmABF2* (GRMZM2G479760), were analyzed in WT, *ZmCaM2*-overexpressing lines (OE1 and OE2) and CRISPR/Cas9-*ZmCaM2* mutant lines (C1 and C2). (**a**,**b**) The expression levels of ABA-responsive genes *ZmRAB18* (GRMZM2G098750) and *ZmABF2* (GRMZM2G479760) under 15 µM ABA treatment at 0 h and 6 h, respectively. Expression level of WT at 0 h was set to 1.0. Relative expression levels were calculated using the 2^−△△CT^ method. The data were the average (±SD) of three independent experiments. The significance analysis compared with WT was performed using one-way ANOVA (** *p* < 0.01). Bars indicate standard error of the mean.

Meanwhile, the expression levels of ABA-responsive genes (*ZmRAB18* and *ZmABF2*) [47] were evaluated in the WT, *ZmCaM2* overexpressing lines, and CRISPR/Cas9-*ZmCaM2* mutant lines under 15 µM ABA treatment at 0 h and 6 h, respectively. The transcript levels of *ZmRAB18* and *ZmABF2* were not significantly different among the WT, OE1 and OE2, and the C1 and C2 lines under ABA treatment for 0 h (Figure 7a,b). However, the transcript levels of *ZmRAB18* and *ZmABF2* were significantly higher in the C1 and C2 lines compared to that of the WT after ABA treatment for 6 h, and the OE1 and OE2 lines exhibited opposite results (Figure 7a,b).

## 3. Discussion

Drought constitutes a major abiotic stress in agriculture, often leading to decreased crop productivity or total crop failure, thereby posing substantial challenges to farmers’ livelihoods [48,49]. Exploring drought tolerance genes and understanding their functions are critical for improving maize drought tolerance. Notably, *CaMs* play essential roles in regulating physiological responses to drought stress in plants [48]. However, the function of *CaMs* in maize remains poorly understood. In this study, we isolated a CaM gene from maize, designated *ZmCaM2*. Evolutionary analysis revealed high homology between ZmCaM2 and OsCaM2, and sequence alignment showed that *ZmCaM2* contains four conserved EF-hand domains (Figure 1). Moreover, ZmCaM2 specifically binds Ca^2+^ and is localized in the nucleus and membrane (Figure 3). Functional analysis indicates that overexpression of *ZmCaM2* reduces maize tolerance to drought stress, whereas functional loss of *ZmCaM2* enhances maize tolerance to drought stress (Figure 4). Further analysis indicated that *ZmCaM2* negatively regulates drought tolerance by modulating multiple physiological parameters and the ABA signaling pathway (Figure 5–7). These results offer valuable insights into the role of *ZmCaM2* in modulating maize drought tolerance via calcium ion binding and the ABA signaling pathway.

Numerous Ca^2+^ binding proteins have been demonstrated to be localized in the cytoplasm, vacuole, cell membrane or the nucleus [50]. In this study, we demonstrated that ZmCaM2 specifically binds to Ca^2+^ and localizes to both the nucleus and cell membrane (Figure 3). Therefore, *ZmCaM2* may directly or indirectly regulate the expression of downstream target genes by binding calcium ions, thereby reducing antioxidant enzyme activity and ABA content while increasing ROS levels, thus diminishing maize tolerance to drought stress.

Several lines of evidence indicate that CaMs play critical roles in mediating responses to drought stress. For example, *OsMSR2* positively regulates drought and salt tolerance [51]. Overexpression of *TaCAM2-D* in *Arabidopsis* enhances tolerance to both drought and salt stress [52]. Plants overexpressing *EcCaM* show enhanced tolerance to drought stress [44]. *MtCaMP1* improves drought tolerance in plants by reducing the accumulation of H_2_O_2_ and MDA [53]. In contrast, *ZmCaM2* negatively regulates drought tolerance in maize (Figure 4), suggesting that it functions as a negative regulator of the drought stress response.

ROS production is essential for protecting plants from various abiotic stresses; however, excessive ROS accumulation can lead to cellular damage [54]. Plants have evolved diverse enzymatic and non-enzymatic antioxidants to scavenge excess ROS and maintain ROS homeostasis [55]. Extensive studies have suggested that CaMs are involved in modulating ROS-related signal transduction [56]. Overexpression of *MsCML46* enhances tolerance to freezing, drought, and salt stresses in tobacco by enhancing antioxidant enzyme activities, and reducing ROS accumulation [57]. Plants overexpressing *ShCML44* show higher antioxidant enzyme activities and lower ROS levels, resulting in enhanced tolerance to abiotic stresses [58]. Following 10 d of drought stress treatment, *ZmCaM2* overexpressing lines showed significantly lower SOD and POD activities, ABA and Pro contents, and RWC, while MDA and ROS content was significantly elevated. In contrast, the CRISPR/Cas9-*ZmCaM2* mutant line exhibited enhanced SOD and POD activities, increased ABA and Pro contents, and reduced MDA and ROS accumulation (Figure 5). These results suggest that *ZmCaM2* functions as a negative regulator of drought tolerance via modulation of ROS homeostasis.

The plant hormone ABA is a key signaling molecule that mediates plant responses to various stresses [59]. Many pieces of evidence demonstrated that Ca^2+^ sensors participate in ABA-mediated signaling pathway [40]. Overexpression of *OsMSR2* in *Arabidopsis* heightened ABA sensitivity and conferred enhanced tolerance to both high salinity and drought stress [51]. *AtCML24* was found to be involved in ABA signaling pathway [60]. *AtCML42* mutants show enhanced tolerance to drought stress through ABA-dependent signaling pathway [61]. *SlCML39* negatively regulates plant tolerance to high temperature stress in an ABA-dependent manner [62]. Under ABA treatment, the primary root length of *ZmCaM2* overexpressing lines was significantly longer than that of the WT and CRISPR/Cas9-*ZmCaM2* mutant lines, both at the seedling stage and during germination (Figure 6). Therefore, *ZmCaM2* mediates the maize response to drought stress via the ABA signaling pathway. In contrast, our previous research demonstrated that homologous *ZmCaM2-1* gene does not mediate any response via the ABA signaling pathway [63], suggesting that different members of the *CaM* gene family fulfill distinct roles in maize. Further exploration of the functions of *CaM* in maize is essential.

## 4. Materials and Methods

### 4.1. Plant Materials, Growing Conditions, and Treatments

The maize inbred lines B73 and B104 were provided by the Maize Breeding Innovation Team of Jilin Agricultural University. Seeds were sown in germination boxes and cultivated under controlled conditions at 28 °C with a 16 h light/8 h dark photoperiod until the V3 stage, with soil moisture maintained at 80% of field capacity. At the V3 stage, seedlings were subjected to drought and hormonal treatments in the cultivation trays, including irrigation with 20% PEG6000 until the soil reaches saturation, foliar spraying with 200 µM ABA and place the cultivation trays in a sealed incubator, or watering as a control. Leaf samples (one-quarter sections from equivalent regions showing uniform growth) were collected from three independent plants at 0, 2, 6, 12, and 24 h post-treatment and stored at −80 °C. The expression levels of *ZmCaM2* were analyzed using qRT-PCR, and all primers used are listed in Table S1.

### 4.2. RNA Extraction and qRT-PCR

A total of 1 µg RNA was extracted using Trizol reagent (Tiangen, Beijing, China) and subsequently reverse-transcribed into cDNA using a TOYOBO reverse transcription kit (TOYOBO, Shanghai, China). qRT-PCR was performed on a QuantStudio 3 instrument (Thermo, Waltham, MA, USA), with ZmActin (GRMZM2G126010) serving as the internal control. Relative gene expression was calculated using the 2^−ΔΔCT^ method [64]. Each experiment included three independent biological replicates. All primer sequences are listed in Table S1.

### 4.3. ZmCaM2 Cloning and Sequence Analysis

The full-length sequence of *ZmCaM2* was cloned from leaves of the inbred maize line B73 using reverse transcription PCR (RT-PCR). Primer sequences are listed in Table S1. Homologous amino acid sequences of *ZmCaM2* were identified by BLAST (v2.17.0) searches against the NCBI database, and phylogenetic analysis was performed using MEGA 7.0. Molecular weight (MW), pI, and GRAVY of *ZmCaM2* were predicted using the ExPASy ProtParam tool (http://web.expasy.org/protparam/, accessed on 3 October 2025).

### 4.4. Subcellular Localization

The full-length CDS of *ZmCaM2* was inserted into the pCAMBIA1302 vector in-frame with GFP using the Seamless Cloning Kit (Beyotime, Shanghai, China), with primer sequences provided in Table S1. Agrobacterium-mediated transformation was used to introduce either the pCAMBIA1302-GFP (35S::GFP) plasmid or the pCAMBIA1302-ZmCaM2-GFP (35S::ZmCaM2-GFP) construct into *Nicotiana benthamiana* plants. Following transformation, plants were maintained in the dark at 22 °C for 16–24 h. GFP fluorescence was visualized using a confocal laser scanning microscope (Leica, Frankfurt, Germany) with excitation at 488 nm.

### 4.5. Prokaryotic Expression Analysis of ZmCaM2 and Ca^2+^ Binding Assay

The CDS region of *ZmCaM2* was cloned into the PET-29b vector via seamless cloning, with all primer sequences provided in Table S1. The recombinant plasmid was subsequently transformed into the *E. coli* BL21 (DE3) prokaryotic expression strain. Recombinant protein was purified using a His-tag Protein Purification Kit (LABLEAD, Beijing, China). For the Ca^2+^-binding assay, purified protein was incubated with either 10 mM CaCl_2_ or 10 mM EGTA.

### 4.6. Transgenic Plant Generation and Drought Tolerance Identification

The CDS of *ZmCaM2*, containing *BamH* I and *Spe* I restriction sites, was cloned into the pCAMBIA3301-UBI vector using T4 DNA ligase (TaKaRa, Beijing, China) to generate the pCAMBIA3301-UBI-ZmCaM2 recombinant plasmid. This plasmid was introduced into the inbred maize line B104 via Agrobacterium-mediated transformation, and T3 transgenic plants were identified by BAR strip assay and qRT-PCR.

The CRISPR/Cas9-*ZmCaM2* mutant lines were generated through targeted knockout. A 20 bp sgRNA was inserted into the pegCas9PUB-B vector, which was subsequently introduced into B104 maize via *Agrobacterium*-mediated transformation. Homozygous mutant lines were confirmed by sequencing.

For drought tolerance evaluation, to ensure uniform soil moisture, we placed the WT B104, *ZmCaM2* overexpression (OE1 and OE2), and CRISPR/Cas9-*ZmCaM2* (C1 and C2) maize lines from the V3 stage into planting trays, maintaining soil moisture at 80% of field capacity. Subsequently, the V3 stage seedlings of *ZmCaM2* overexpressing lines, CRISPR/Cas9-*ZmCaM2* mutants, and WT lines were subjected to drought stress by withholding water for 10 d until the soil moisture reached 35% of field capacity. The seedlings from WT, *ZmCaM2* overexpressing lines, and CRISPR/Cas9-*ZmCaM2* mutant lines were selected, washed, and dried at 105 °C until they reached a constant weight, after which they were weighed. Subsequently, continue watering for 3 d to maintain soil moisture at 80%. Drought tolerance images of WT B104, *ZmCaM2* overexpressing lines, and CRISPR/Cas9-*ZmCaM2* lines were captured at the V3 stage, on days 10 of drought treatment and days 3 of rewatering with a Nikon D7000 camera (Nikon, Tokyo, Japan).

### 4.7. Physiological Indicators Measurement

Physiological indices of WT, *ZmCaM2* overexpressing lines and CRISPR/Cas9-*ZmCaM2* mutant lines were measured after 10 d of drought treatment. About 0.5 g of plant leaves were utilized for the determination of RWC, MDA, Pro, ABA and ROS content, and the activities of SOD and POD. RWC of leaves was measured according to the previously described method [65]. MDA content was determined using the thiobarbituric acid-based method [66]. Pro content was determined using the indophenol III colorimetric method [67]. The ABA content was determined using the plant abscisic acid ELISA detection kit (ELK, Wuhan, China), according to the manufacturer’s guidelines. Following the manufacturer’s instructions (KETE, Wenzhou, China), ROS were extracted using the Plant ROS ELISA Kit. ABA and ROS content were measured at 450 nm using a full-wavelength enzyme-labeling apparatus (HBS-ScanY, Shanghai, China). SOD activity was measured using the nitrotetrazolium blue chloride method [68]. POD activity was determined according to the previously described method [69]. The absorbance values of MDA, SOD, and POD were measured using a spectrophotometer (INESA, Shanghai, China). The experiment was performed using three biological replicates from three independent plants.

### 4.8. ABA Stress Treatment

The seeds of WT, *ZmCaM2* overexpressing lines (OE1 and OE2), and CRISPR/Cas9-*ZmCaM2* mutant lines (C1 and C2) were sterilized and placed on wet filter paper at 28 °C for 3 d until the taproot reached about 3 cm. The seedlings were transferred into Hoagland solution containing 0 µM or 15 µM ABA for 10 d, and the primary root length was measured and calculated. The experiment was performed with three independent biological repetitions. The three-leaf stage seedlings were treated with 15 µM ABA, and the samples were collected at 0 h and 6 h, respectively.

For the seed germination experiment under ABA stress, we disinfected seeds from the WT, *ZmCaM2* overexpressing lines, and CRISPR/Cas9-*ZmCaM2* mutant lines, followed by treatment with 0 μM or 10 μM ABA for 4 d, and the primary root length was measured and calculated.

### 4.9. Statistical Analysis

The statistical experiments were performed with three biological replicates. All data were analyzed using GraphPad Prism 9.0 software. The significant differences between samples were analyzed using one-way ANOVA (* *p* < 0.05, ** *p* < 0.01) and bars indicate the standard error of the mean.

## 5. Conclusions

**Figure 8:**
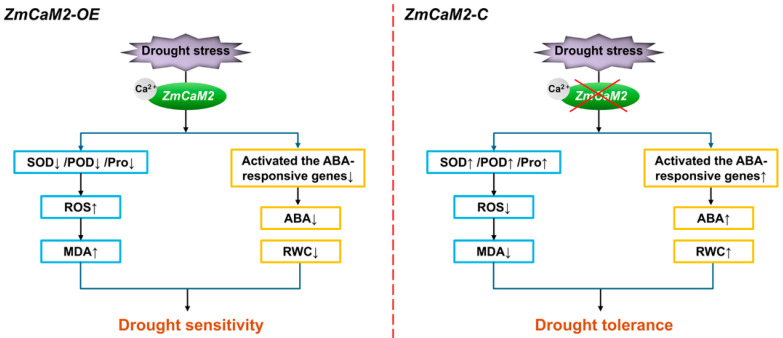
A proposed model of *ZmCaM2* in response to drought stress.

In our work, we focused on drought tolerance at the seedling stage and found that *ZmCaM2* may reduce the tolerance of maize to drought stress by binding Ca^2+^ and relying on the ABA signaling pathway to suppress antioxidant enzyme activity, decrease RWC and ABA content, and increase ROS accumulation (Figure 8). However, its effects on agronomic traits and yield under field drought conditions, as well as its practical value in breeding, require long-term evaluation in larger populations. To mitigate yield losses under climate change, assessing the potential application of *ZmCaM2* in drought- and stress-resilient breeding will remain a major focus of our future research. This line of work has already been incorporated into our genome-assisted breeding strategy.

## Data Availability

All data generated or analyzed during this study are included in this published article and its Supplementary Materials files.

## Supplementary Materials

The following supporting information can be downloaded at: https://www.mdpi.com/article/10.3390/plants14233656/s1, Figure S1: Detection of transgenic maize; Figure S2: Detection of mutant maize; Table S1: RT-qPCR primers used in this study.

## Abbreviations

The following abbreviations are used in this manuscript:

qRT-PCR	Quantitative real-time polymerase chain reaction
ABA	Abscisic acid
ROS	Reactive oxygen species
Ca^2+^	Calcium ions
CaM	Calmodulin
CMLs	Calmodulin-like proteins
CDPKs	Calcium-dependent protein kinases
CBLs	Calcineurin B-like proteins
CCaMKs	Calcium and calmodulin -dependent protein kinases
GFP	Green fluorescent protein
WT	Wild type
h	Hour
d	Day
V3	Third-leaf stage
CDS	Coding sequence
RWC	Relative water content
SOD	Superoxide dismutase
POD	Peroxidase
MDA	Malondialdehyde
Pro	Proline
PI	Isoelectric point
GRAVY	Grand Average of Hy-dropathicity
MW	Molecular weight
RT-PCR	Reverse transcription PCR
