# Supplementary material for: Zea Maize Calmodulin (ZmCaM2) Regulates Drought Tolerance in Corn Plants Through an Abscisic Acid-Dependent Signaling Pathway
Source: Plants (Basel). 2025 Nov 30;14(23):3656. doi: 10.3390/plants14233656 (PMC12693906; doi:10.3390/plants14233656)
Supplement: Supplementary file 1 [file plants-14-03656-s001.zip › Supplementary Information .pdf]

Table S1. RT-qPCR Primers used in this study

Gene ID	Forward primer sequence (5'–3')	Reverse primer sequence (5'–3')
ZmCaM2	TCGCCATGGCGGACCA	TCCGGCTCACAGGATCACG
pCAMBIA1302-ZmCaM2	TGACCATGGTAGATCTATGGCGGACCAGCTTAGC	CTTCTCCTTTACTAGTTGACAGGATCACGCATTTCTGG
PET-29b-ZmCaM2	TTAGAAGGAGATATACATATGATGGCGGACCAGCTTAGC	ACGGAGCTCGAATTCGGATCCTGACAGGATCACGCATTTCTGGC
pCAMBIA3301-UBI-ZmCaM2	CGGGATCCATGGATTACAAGGATGACGACGATAAGATGGCGGACCAGCTTAGC	ACTAGTTCACAGGATCACGCATTTCTGG
M1830(493)	TTGAAGACTTATGGTCTGAAGC	ACGAACTCGTCGTAGTTGATG
pU6-T1	CTCCGTTTTACCTGTGGAATCG	CATCACGGTTCCGAGCTCCTCGGCAGCCAAGCCAGCA
gRNA-T1	CGGAGGAAAATTCCATCCAC	AGGAGCTCGGAACCGTGATGGTTTTAGAGCTAGAAAT
Pps	AGCGTGGGTCTCGCTCGACGCGTATCCATCCACTCCAAGCTC	TTCAGAGGTCTCTACCGACTAGTATGGAATCGGCAGCAAAGG
SP	GCGGTGTCATCTATGTTACTAG	TGCAATAACTTCGTATAGGC
ZmABF2-q	TTGTGCCTCCACTGCAATTCGG	TTACAAGTCGTCTCCCTCCATCTTCC
ZmRAB18-q	GTGGTGGGTTGAGGGGAAGGAAGC	ACGCCATCGCCGTTGAGCCT
ZmActin	CACTTCCTCATGCTATTCTCCG	GCTTCTCCTTTATGTCCCTGAC

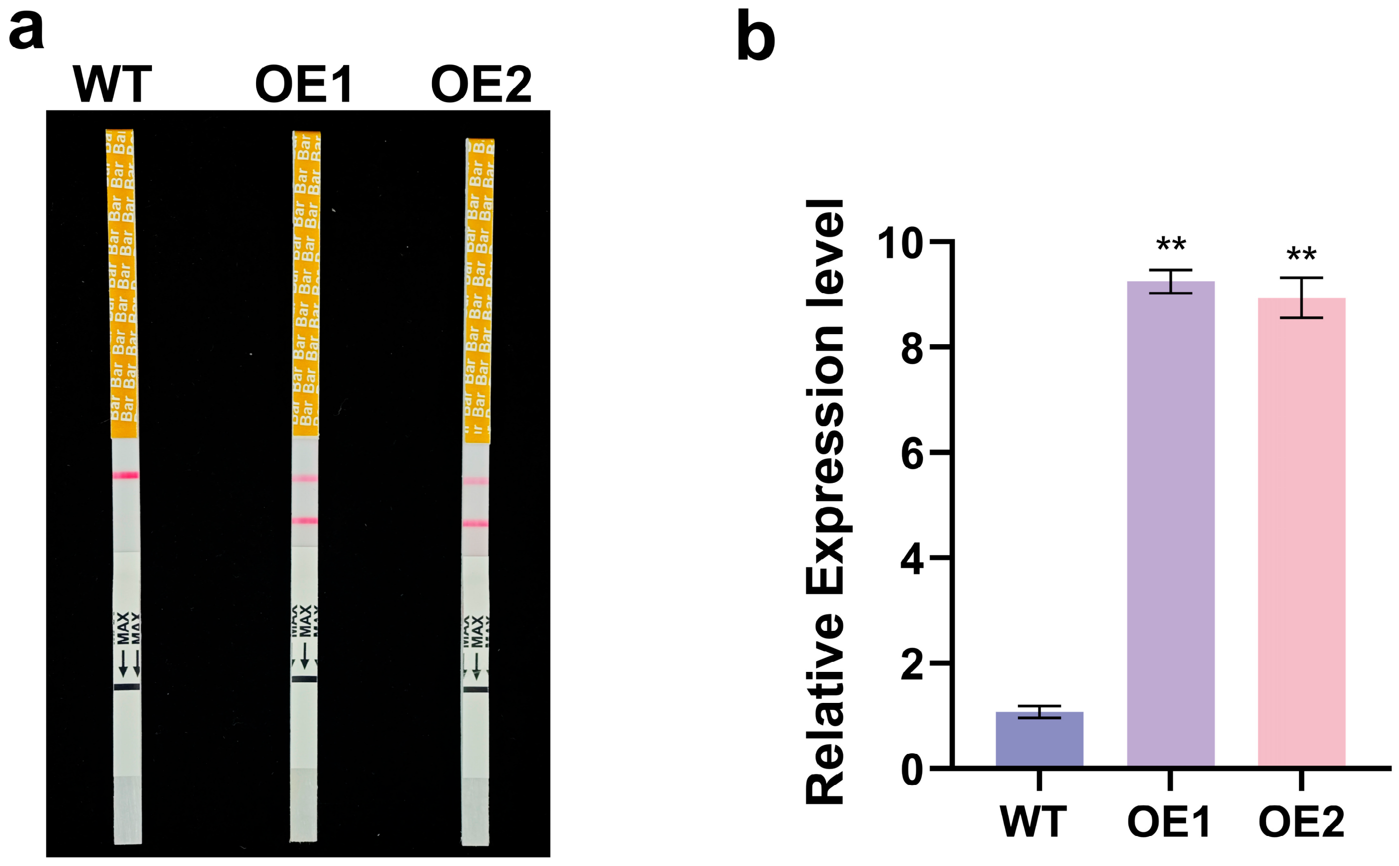

Figure S1. Detection of transgenic maize. (a) Bar test strip detection. (b) Expression detection of the *ZmCaM2* overexpressed B104 maize lines. The significance analysis compared with WT was performed using one-way ANOVA (***P* < 0.01). Bars indicate standard error of the mean. The experiment was performed using three biological replicates.

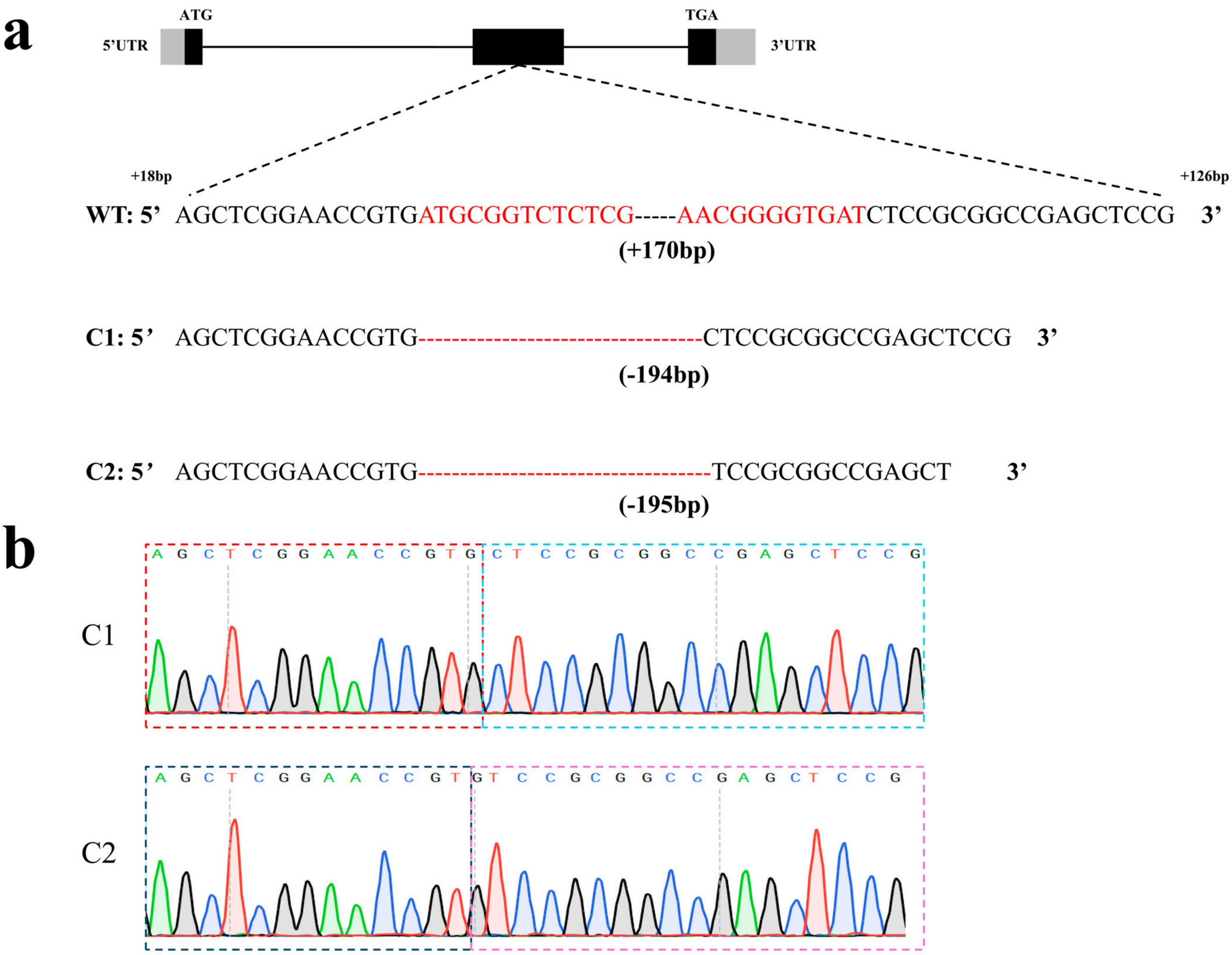

Figure S2. Detection of mutant maize. (a) Schematic diagrams of CRISPR/Cas9-*ZmCaM2* mutant lines generated using CRISPR/Cas9–mediated genome editing. (b) The CRISPR/Cas9-*ZmCaM2* mutant lines were identified using Sanger sequencing compared with WT.
